# Malignant transformation of calcifying epithelial odontogenic tumour: A systematic review

**DOI:** 10.4317/jced.62804

**Published:** 2025-09-01

**Authors:** Elton Fernandes Barros, Esllen Carla Ferreira de Araújo Vasconcelos, Ellen da Silva Gonçalves, Renally Bezerra Wanderley Lima, Cassiano Francisco Weege Nonaka, Hellen Bandeira de Pontes Santos

**Affiliations:** 1 Department of Dentistry, State University of Paraíba- UEPB, Campina Grande, PB, Brazil; 2 Department of Dentistry, Faculty of Nursing and Medicine Nova Esperança- FACENE, João Pessoa, PB, Brazil; 3 Professional Master’s Program in Family Health, Faculty of Nursing and Medicine Nova Esperança- FACENE, João Pessoa, PB, Brazil; 4 Department of Restorative Dentistry, Federal University of Paraíba- UFPB, João Pessoa, PB, Brazil

## Abstract

**Background:**

The calcifying epithelial odontogenic tumor (CEOT) is a rare benign epithelial odontogenic neoplasm. Some cases of CEOT may undergo malignant transformation, whose characteristics are still poorly known. This study aimed to perform a systematic review of CEOT cases with malignant transformation.

**Material and Methods:**

This systematic review followed the preferred reporting items for systematic reviews and meta-analyses (PRISMA) and was registered with PROSPERO (CRD42021285981). Searches for full-text articles on histopathologically confirmed CEOT cases with malignant transformation were performed in different databases (PubMed/ MEDLINE, Embase, Scopus, Web of Science, LILACS, SciELO, Google Scholar, Open Grey, and CAPES Dissertation and Thesis Catalog) without year of publication or language restrictions. A qualitative descriptive and risk of bias analysis were performed.

**Results:**

Nine cases were included, with a mean age of 59.44 (±17.07) years and a slightly higher frequency in males (55.6%). The mandible (88.9%) was the most affected site, with predominance of the mixed imaging pattern (77.8%). Histopathologically, the clear cell variant, intense mitotic activity, presence of cellular atypia, and high Ki-67 immunoexpression were the predominant findings. Isolated surgery (44.4%) was the most common treatment and recurrence of CEOT before malignant transformation was observed in five cases (55.6%). CEOT with malignant transformation recurred in three cases (33.3%). Most cases had a positive outcome (77.8%), with remission of the disease.

**Conclusions:**

This systematic review determined the clinicopathological profile of histopathologically confirmed cases of CEOT with malignant transformation and synthesized some characteristics that can assist in the diagnosis and appropriate therapeutic approach of this rare neoplasm.

** Key words:**Systematic review, Odontogenic tumors, Clinicopathologic features, Treatment, Prognosis.

## Introduction

Calcifying epithelial odontogenic tumor (CEOT), also known as Pindborg tumor, is a rare benign epithelial odontogenic neoplasm that accounts for approximately 1% of all odontogenic tumors [1-3]. However, although considered a benign and rare tumor, CEOT can exhibit local aggressive behavior and cases of malignant transformation with distant metastases have been reported in the literature [4-7].

Clinically, CEOT generally appears as an intraosseous (central) asymptomatic mass of expansive and slow growth. The extraosseous (peripheral) presentation is less common and is limited to the soft tissue where it appears as a nodular lesion in the region of the gingival mucosa. Usually less aggressive than the intraosseous presentation [4,6,8,9]. The most affected anatomical sites are the posterior mandible and the posterior maxilla. There is a higher prevalence in the age range from the third to the fourth decade of life and a slight female predilection [3,5,6,8,10,11]. Radiographically, CEOT exhibits different patterns of radiodensity ranging from unilocular to multilocular tumors with well-defined or diffuse margins and radiopaque calcifications of varying sizes and opacities [2,8,10,12,13].

Histopathologically, CEOT consists of trabeculae and islands of polyhedral epithelial cells with well-delineated abundant eosinophilic cytoplasm; intercellular bridges are observed in some tumors. Considering its local invasive growth pattern, CEOT exhibits nuclear pleomorphism, anisocytosis, anisonucleosis, hyperchromatism and, generally, very few or absent mitotic Figures, except for cases of malignant transformation in which cell proliferation markers, such as Ki-67, tend to be altered [2,4,6,7,10,13,14]. The presence of Liesegang rings, which are concentric rings of calcified eosinophilic hyaline material, is characteristic of this tumor. Furthermore, histological variants of CEOT have been reported in the literature, with the clear cell variant being one of the most aggressive [3,8-10].

An early diagnosis of CEOT is very important for favoring a less invasive approach and a better prognosis [4,7,15]. However, this diagnosis poses a great challenge because of the characteristics of these tumors [7,8]. The prognosis of most tumors diagnosed as CEOT tends to be favorable, except for cases of malignant transformation. Thus, the therapeutic approach is established on an individual basis considering clinical, radiographic, and histopathological characteristics. Treatment consists of surgical removal; in cases of malignant transformation, radiotherapy and/or chemotherapy are commonly added. The recurrence rate of CEOT ranges from 10 to 15% [2,4,6,7,16]. In addition, long-term follow-up of these patients is essential [7,10,11].

Therefore, the aim of this study was to perform a systematic review of histopathologically confirmed cases of CEOT with malignant transformation reported in the literature, focusing on demographic, clinical, imaging, histopathological, immunohistochemical, therapeutic, and prognostic features, and thus synthesizing characteristics that can assist in the diagnosis and appropriate therapeutic approach.

## Material and Methods

This systematic review was conducted according to the 2020 Preferred Reporting Items for Systematic Reviews and Meta-Analyses (PRISMA) guidelines [17] and the methodological criteria were registered in the International Prospective Register of Systematic Reviews (PROSPERO) (CRD42021285981). The study was based on the following research question: What are the demographic, clinical, imaging, histopathological, immunohistochemical, therapeutic, and prognostic characteristics of calcifying epithelial odontogenic tumor with malignant transformation?

- Eligibility criteria

Observational studies, longitudinal studies, case reports and case series reporting histopathologically confirmed CEOT with malignant transformation were included. Experimental studies, *in vitro* studies, letters to the editor and review articles were excluded, unless these studies reported cases of CEOT with malignant transformation that had sufficient clinical, imaging, and histopathological information.

- Information sources and search strategy

Searches of full-text articles were performed in different electronic databases (PubMed/ MEDLINE, Embase, Scopus, Web of Science, LILACS, and SciELO) and in the grey literature (Google Scholar, Open Grey, and CAPES Dissertation and Thesis Catalog), without year of publication or language restrictions. These systematic searches were performed by two of the authors, previously calibrated. The last search in the databases was conducted in April 2025. Controlled descriptors and free search terms were used as the search strategy for the chosen databases: (“calcifying epithelial odontogenic tumor” OR “calcifying epithelial odontogenic tumour” OR “Pindborg tumor” OR “Pindborg tumour” OR CEOT) AND (malignant OR malignancy OR transformation OR carcinoma) (supplementary material).

- Study selection

The search strategy retrieved 801 publications, which were evaluated independently by two reviewers. The Rayyan tool was used for selection of the studies and removal of duplicates. The studies were selected in two steps. First, two reviewers (EFB and ECFAV) independently screened the titles and abstracts of all articles identified. Studies that did not meet the eligibility criteria mentioned above were excluded. In the second step, the same two reviewers applied the eligibility criteria to the full text of the studies. In the case of disagreement that could not be resolved between the two reviewers, a third reviewer (HBPS) was consulted to reach consensus. Finally, nine studies were included.

The full text of the article was read if there was not enough information in the titles and abstracts and the study was included when it met the eligibility criteria.

- Data collection process

After selection of the articles, the following data were extracted using a standardized form and entered into an Excel spreadsheet: authors’ name, year of publication, demographic data, number of cases, patient age and sex, and duration and anatomic location (maxilla/mandible) of the tumor. Clinical characteristics (intraosseous/extraosseous), imaging features (radiolucent/radiopaque/mixed/hypodense/hyperdense), locularity (unilocular/ multilocular), definition of tumor margins (well defined/poorly defined), and tumor size (largest diameter in cm) were also obtained. In addition, the following data were collected: histopathological features (predominant morphological pattern, presence of atypia), including histological variants (CEOT with clear cells, CEOT with Langerhans cells, CEOT with microcysts, non-calcifying Langerhans cell-rich variant of CEOT, CEOT associated with another epithelial odontogenic tumor, and CEOT with myoepithelial cells), immunohistochemical markers (Ki-67, p53, cytokeratins), treatment performed (surgery/radiotherapy/chemotherapy), metastasis (yes/no and location), recurrence (yes/no), duration of follow-up (in months), and patient outcome (death/remission/alive with disease).

- Risk of bias assessment

The Joanna Briggs Institute – University of Adelaide critical appraisal tool for case report studies [18] was used for the risk of bias assessment by two authors. Disagreements were resolved by a third author. The following parameters were used for evaluation: clear description of the patient’s demographic characteristics and medical history; clear description of the current clinical condition; clear description of assessment methods, diagnostic tests and results; clear description of intervention or therapeutic procedure; clear description of the post-intervention clinical condition; identification of adverse events, and lessons provided by the case report. For each parameter, the article was classified as “yes”, “no’, “unclear”, or “not applicable”.

- Synthesis methods

The data extracted from the studies that met the inclusion criteria were evaluated regarding homogeneity. Qualitative descriptive analysis of the data was performed using the Statistical Package for the Social Sciences (version 22.0; IBM Corp., Armonk, NY, USA).

## Results

- Study selection

**Figure 1:**
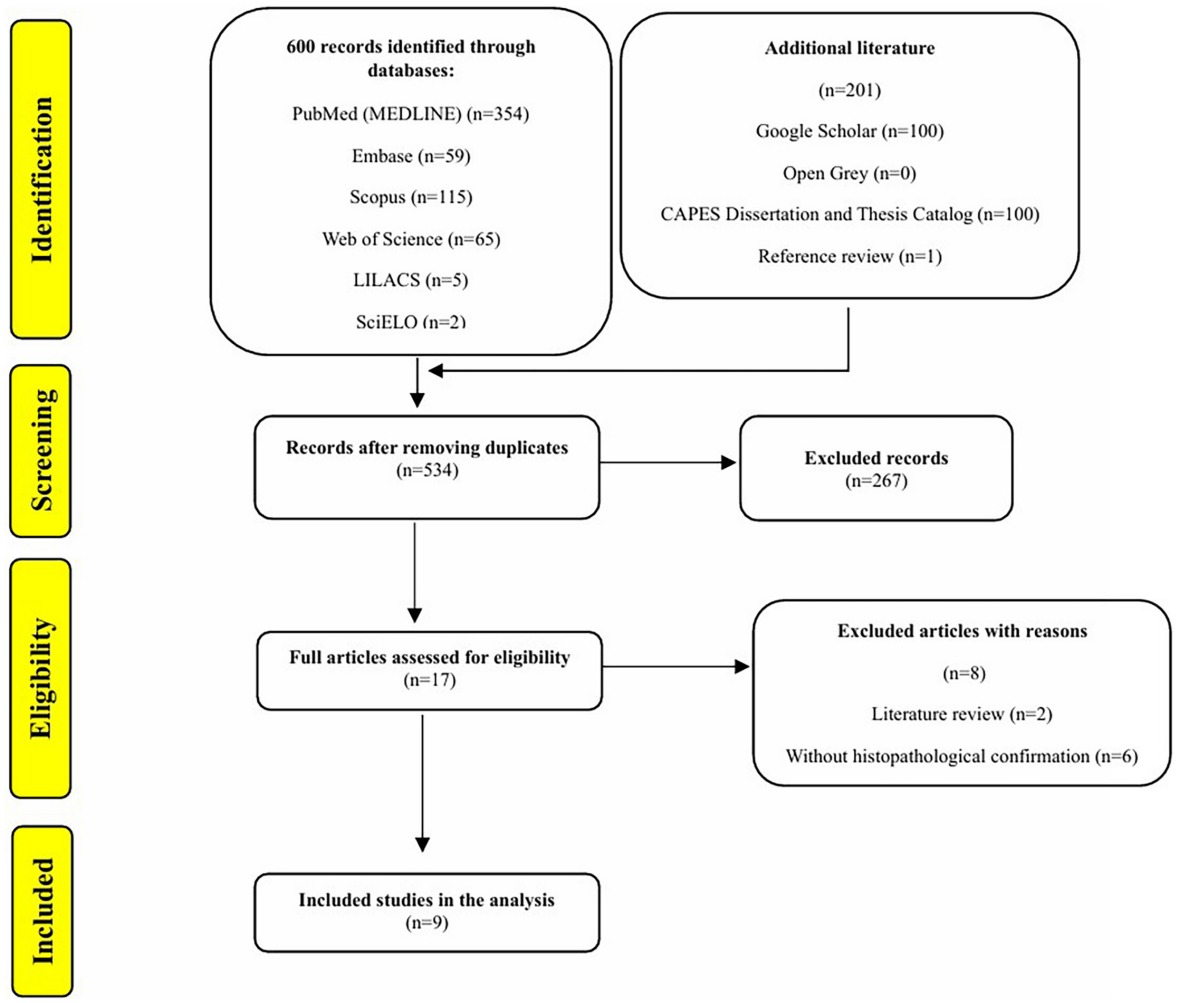
Flow diagram of literature research and study selection process (adapted from Preferred Reporting Items for Systematic reviews and Meta-Analyses) (Page *et al*. [17]).

Figure 1 illustrates the study selection process. The search strategy retrieved 801 publications; 17 were evaluated based on the eligibility criteria. Among the studies analyzed, there were nine histopathologically confirmed cases of CEOT with malignant transformation [7,15,19-25].

- Results of syntheses

The demographic, clinical, and imaging characteristics are shown in  Table 1. Japan was the country with the largest number of cases (n = 3) [19-21]. The mean age of the nine cases was 59.44 (±17.07) years [7,15,19-25]. There was a slightly higher frequency of males (55.6%) compared to females (44.4%). The mandible was the most affected site, observed in eight cases (88.9%) [7,15,19-24], and the intraosseous clinical presentation corresponded to 100% of cases. Regarding imaging features, the mixed pattern was found in seven cases (77.8%) [7,15,20-23,25]. The percentage of unilocular and multilocular tumors was similar and there was a higher frequency of cases with well-defined margins (66.7%).

The histopathological features are presented in  Table 2. The histological variant of CEOT with clear cells was observed in four cases [7,22,24,25]. The presence of intense mitotic activity and cellular atypias were found to be important characteristics in the nine casesof malignant transformation [7,15,19-25], as was the evident immunoexpression of the cell proliferation marker Ki-67, which was observed in 9.6% to 42% in the cases. Furthermore, malignant transformation occurred during tumor recurrence in six cases [7,15,19-21,23]. A hybrid nature of the tumor was reported in two cases [15,25].

Regarding therapeutic and prognostic characteristics ( Table 3), surgery, radiotherapy, and/or chemotherapy were the approaches used, with isolated surgery being the most common treatment (44.4%) [21,22,24,25]. Recurrence of the classic CEOT before malignant transformation was observed in five cases (55.6%) [15,19-21,23]. CEOT with malignant transformation recurred in three cases after treatment (33.3%) [15,20,22]. Metastasis was found in six cases (66.7%) [7,15,19,20,22,23], with lung metastases being the most common. The longest period of follow-up was 60 months [7], with a mean duration of 26.6 (±20.07) months. Regarding the outcome of the included cases, remission of the disease was reported in seven cases (77.8%) [7,19,21-25], one patient was alive with disease (11.1%) [20], and one had died (11.1%) [15].

- Risk of bias

**Figure 2:**
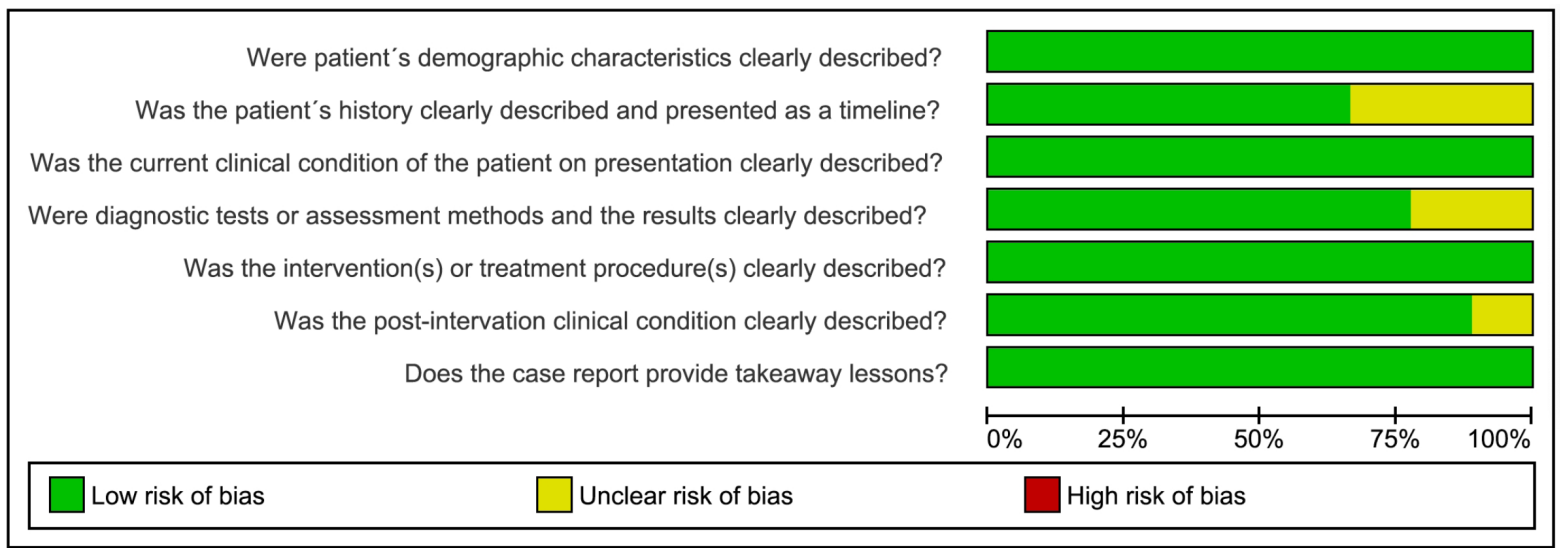
Risk of bias of included case reports (prepared through the Review Manager 5.4).

Five studies had a low risk of bias [7,20,21,23,24], while four studies were classified as unclear risk of bias in some parameters [15,19,22,25]. The parameter that most increased the risk of bias was “Was the patient’s history clearly described and presented as a timeline?” Three studies did not report sufficient information or in a timeline [15,22,25]. In addition, two studieshad an unclear risk of bias in the parameter “Were diagnostic tests or assessment methods and the results clearly described?” [15,19], and one study in the parameter “Was the post-intervention clinical condition clearly described?” [22]. Figure 2 summarizes the risk of bias assessment.

## Discussion

Although CEOT, or Pindborg tumor, is a rare epithelial benign odontogenic neoplasm, analysis of the included studies and case reports of CEOT with malignant transformation demonstrates the highly variable biological behavior and high aggressiveness of this tumor [2,4,7], including even cases of metastatic tumors reported by Basu *et al*. (1984), Veness *et al*. (2001), Nagahama *et al*. (2002), Kawano *et al*. (2007), Demian *et al*. (2010), and Tabaksert *et al* (2021). Thus, the clinical, imaging, and histopathological characteristics of this tumor are essential for an adequate diagnosis and treatment in these cases [4,7].

Clinically, CEOT can be divided into two presentations. The intraosseous (central) is the most common among benign and malignant cases of CEOT reported here. It appears as a slow-growing, expansive, asymptomatic intraosseous mass that causes cortical bone expansion, tooth migration, mobility and rotation, and root resorption. The extraosseous (peripheral) presentation is rarer and less aggressive compared to the central and is limited to soft tissue only [4,6,26]. The intraosseous predominated among the CEOT cases with malignant transformation analyzed. As observed in cases of benign CEOT, the mandible was the most affected anatomical site. Regarding sex, there was a slightly higher frequency of CEOT cases with malignant transformation in males (55.6%), which is the opposite of the cases of benign lesions that commonly have a slight female predilection [5,11].

Regarding imaging findings, mixed radiodensities are observed in most benign cases of CEOT depending on the stage of development. Commonly, imaging reveals a unilocular or multilocular lesion with a radiolucent area that exhibits well-defined or diffuse margins and a radiopaque mass of variable size and opacity; there might be a strong association with unerupted teeth [6,8,10,12,13]. Likewise, in cases of CEOT with malignant transformation, the mixed imaging pattern corresponded to most cases (77.8%) [7,15,20-23,25]; the tumors showed similar locularity, with well-defined margins. In addition, thinning and erosion of cortical bone were observed, characteristics that are also present in benign tumors [7,11,21].

Histopathologically, CEOT exhibits different architectural patterns that range from small or barely perceptible islands, cords or trabeculae to sheets of polyhedral epithelial cells with well-delineated abundant eosinophilic cytoplasm and evident intercellular bridges [2,6,8,9,14]. Cellular and nuclear atypia is very common in these tumors [2,6], especially in cases of malignant transformation. In this review, we found intense nuclear and cellular pleomorphism, as well as atypical mitotic Figures such as tripolar Figures in the case reported by Cheng *et al*. (2002). Benign CEOT commonly exhibits Ki-67 expression less than 2% [4], while in the cases of malignant transformation analyzed here, the lowest percentage was 9.6% reported by Cheng *et al*. (2002) and the highest was 42% reported by Demian *et al*. (2010). There was also expression of other immunohistochemical markers. According to El-Naggar *et al*. (2017), the absence or small number of mitotic Figures, the presence of amyloid protein in the stroma, and low percentages of Ki-67 immunoexpression are characteristics that distinguish the nature of these tumors [9,13]. This was demonstrated in the present study in which mitotic activity and expression of cell proliferation markers (Ki-67) were high in cases of malignant transformation, as well as the absence of amyloid material and calcification in some cases, together with cytological changes and bone and vascular invasion.

An early diagnosis is important in cases of CEOT, especially tumors with a more aggressive local behavior and malignant transformation. The combination of clinical analysis and radiographic and histopathological examination is extremely necessary for an accurate diagnosis and the planning of adequate treatment [2,4,7]. In cases of malignant transformation, clinical and histopathological diagnosis is essential for distinguishing benign, recurrent and malignant tumors in order to guide therapy and follow-up [7,15]. Furthermore, it is important to mention the existence of cases of hybrid tumors such as those reported by Demian *et al*. (2010) and Zhong *et al*. (2010), in which the tumor is composed of a benign and a malignant part in different regions. This fact highlights the importance of histological analysis of the entire tumor in order to avoid misdiagnosis.

The prognosis of CEOT tends to be favorable, except for cases of malignant transformation [5,13]. Within this context, Demian *et al*. (2010) have reported a case of death. The most common surgical techniques for the treatment of clinical presentations of CEOT include enucleation and excision or curettage, generally applied to small tumors. However, in the case of larger tumors, segmental resection may be necessary because of their more destructive and invasive nature. Furthermore, the removal of healthy surgical margins of both bone and soft tissue is essential and must be confirmed by histopathology [2,4,10,11]. In previously reported cases of CEOT with malignant transformation, the treatment modalities were surgery (44.4%), surgery and radiotherapy (22.2%), surgery and chemotherapy (22.2%), and surgery/ radiotherapy and chemotherapy (11.1%) given the aggressiveness of the tumor and the presence of metastasis in many cases, with the lung (33.3%) being the most affected organ [7,15,19,20].

 The recurrence rate of CEOT ranges from 10 to 15% and long-term follow-up must comprise a minimum period of 5 to 10 years [4,7,10]. Before malignant transformation of CEOT, the recurrence was identified in 5 cases (55.6%) [15,19-21,23], which raises an important characteristic to be verified. In the cases of CEOT with malignant transformation, 3 cases (33.3%) recurred after treatment [15,20,22], despite the short follow-up period, the case reported by Tabaksert *et al*. (2021) was the only one with a 5-year follow-up. This is a fact that highlights the importance of meticulous analysis of this feature for future case reports. A positive outcome (remission of the disease) was observed in most cases (77.8%).

## Conclusions

This study identified 9 cases of histopathologically confirmed CEOT with malignant transformation. There was a higher occurrence between the third and eighth decades of life and a slightly higher frequency in males, diverging from benign CEOT cases. Most cases corresponded to the mixed radiographic pattern (77.8%). Histopathologically, there was intense cellular and nuclear pleomorphism, as well as atypical mitotic Figures (Ki-67 ranging from 9.6% to 42%), bone and vascular invasion. Isolated surgery was the most common treatment, sometimes supplemented with radiotherapy and/or chemotherapy, with complete lesion remission in 77.8% of the cases. Recurrence should be a highlighted feature in the analysis of these cases, emphasizing the importance of long-term follow-up.

This systematic review determined the clinicopathological profile of histopathologically confirmed cases of CEOT with malignant transformation and synthesized some characteristics that can assist in the diagnosis and appropriate therapeutic approach of this rare neoplasm.

## Figures and Tables

**Table 1:** Demographic, clinical and imaging characteristics of CEOT cases with malignant transformation.

Authors (year of publication)	Country	Age and sex	Anatomical location	Clinical presentation	Duration of lesion (m)	Size (cm)	Imaging pattern	Locularity	Edges of the lesion
Basu et al. (1984)	England	75M	Mandible	Intraosseous	732	--	Mixed	Multilocular	Well defined
Veness et al. (2001)	Australia	64F	Mandible	Intraosseous	--	--	Mixed	Unilocular	Well defined
Cheng et al. (2002)	United States	83F	Mandible	Intraosseous	--	--	Radiolucent	Multilocular	Well defined
Nagahama et al. (2002)	Japan	56M	Mandible	Intraosseous	--	--	--	--	--
Kawano et al. (2007)	Japan	54M	Mandible	Intraosseous	--	--	Mixed	Unilocular	Well defined
Demian et al. (2010)	United States	45F	Mandible	Intraosseous	12	4,5x4x2,2	Mixed	Multilocular	Ill-defined
Zhong et al. (2010)	China	49M	Maxilla	Intraosseous	12	4x3x3	Mixed	Unilocular	Ill-defined
Tabaksert et al. (2021)	England	31F	Mandible	Intraosseous	5	2,2x3,2	Mixed	Multilocular	Well defined
Aoki et al. (2021)	Japan	78M	Mandible	Intraosseous	--	2,5x2	Mixed	Unilocular	Well defined

Legend: CEOT (calcifying epithelial odontogenic tumor); F (Female); M (Male); m (months). Source: Author.

**Table 2:** Histopathological characteristics of CEOT cases with malignant transformation.

Authors (year of publication)	Histological variant	Morphological pattern of the initial lesion	Morphological pattern of recurrence	IHC
Basu et al. (1984)	CEOT with clear cells	Sheets of polyhedral epithelial cells, with intercellular bridges, with intense cellular and nuclear pleomorphism, as well as multinucleated giant cells (syncytia), in fibrous stroma/ Clear cells/ Positive eosinophilic material (amyloid) for Congo red and presence of Liesegang rings/ Numerous mitotic figures/ Vascular invasion and perineural invasion (inferior alveolar nerve)/ Tumor cells in peripheral sinus and submandibular lymph node cortex (lymph node metastasis).	Recurrent lesion: encapsulated mass within a lymph node, similar structure to the primary tumor, except for the absence of areas of amyloid or mineralization.	Cytokeratin -, carcinoembryonic antigen-, actin -, immunoglobulin classes and light chain types -, alpha-fetoprotein -, B and D cathepsin -, lysozyme -, 1-antitrypsin -
Veness et al. (2001)	--	Sheets and cords of polyhedral epithelial cells with eosinophilic cytoplasm, nuclear hyperchromatism/ No mitosis/ Amyloid material and calcified concentric laminated rings (benign CEOT).	Recurrent lesion after 9 months: lesion more cellularized than the initial/ Apparent mitoses/ Absence of calcification and amyloid material (benign CEOT). Second local recurrence after 15 months: mitoses in tumor cells/ Tumor invasion of small blood vessels/ Absence of calcification and amyloid material/ Surface of the epithelium of the oral mucosa wasn't dysplastic (malignant Pindborg tumor).	--
Cheng et al. (2002)	CEOT with clear cells	Incisional biopsy: cords and islands of eosinophilic polygonal epithelial cells/ Prominent nuclear pleomorphism/ Deposits of hyalinized eosinophilic material within and in the periphery of the tumor islands/ Calcification in some areas of the hyalinized eosinophilic material/Positive staining for Congo red and apple green birefringence in some hyalinized areas. Excisional biopsy: nests of clear cells with eosinophilic cells/ Mild to moderate pleomorphism in clear cells/ Infiltrative growth pattern/ Invasion of bone and blood vessels extending to the surface epithelium/ Nuclear pleomorphism/ Presence of mitotic and tripolar mitotic figures (atypical calcifying epithelial odontogenic tumor with features suggestive of malignity).	No recurrence.	ki-67 (9,6%), Cytokeratin 19+, AE1:AE3+
Nagahama et al. (2002)	--	Oval tumor cells with eosinophilic cytoplasm in a solid pattern, showing mononucleated and multinucleated cells, scarce mitosis figures/ Calcification and positive amorphous material (amyloid) for Congo red (benign CEOT).	First recurrence: histological characteristics similar to the primary tumor (benign CEOT). Second recurrence: similar to the primary tumor, but with a more infiltrative growth pattern/ Reduced number of mitosis figures/ Absence of amyloid material. Third and fourth recurrence: small and large tumor cells with eosinophilic cytoplasm/ Large mononucleated or multinucleated tumor cells with prominent nucleoli and eosinophilic cytoplasm, with lamellar structure/ Abnormal mitosis and necrosis/ Absence of amyloid material and calcification/ Vascular invasion (malignant CEOT). Metastasis in right supraclavicular lymph node.	--
Kawano et al. (2007)	--	April 1995: sheets of polyhedral epithelial cells accompanied by deposition of eosinophilic amyloid material/ Positive for Congo red and apple-green birefringence in the eosinophilic material/ Tumor cells showed slight nuclear pleomorphism, initial diagnosis of CEOT.	April 1996: first recurrence with similar histological characteristics to the primary tumor (benign CEOT). July 1996: second recurrence showing islands of polyhedral epithelial tumor cells with marked pleomorphism/ IHC: Amelogenin +/ Mitotic figures/ Blood vessels with tumor cells in the lumen/ Presence of amyloid-like material (histological findings suggestive of CEOT with malignant transformation). December 1997: third local recurrence with histological characteristics compatible with malignant CEOT. September 2000: lung metastasis - proliferation of polyhedral tumor cells, similar to those of recurrent mandibular lesions/ IHC: Amelogenin +.	ki-67 1995 - 3,5% Jul. 1996 - 10,4% 1997- 13,5% 2000- 13,4%
Demian et al. (2010)	--	Incisional biopsy: islands and sheets of uniform polyhedral cells with well-defined limits and eosinophilic cytoplasm/Amorphous amyloid material, Liesegang rings/ Positive for Congo red and apple-green birefringence (classic CEOT).	March 2004: tumor consisting of two parts, in a dense fibrous stroma, one part with characteristics of benign CEOT, and the other of an odontogenic carcinoma composed of poorly differentiated malignant squamous epithelial cells with eosinophilic cytoplasm/ Extensive pleomorphism/ Hyperchromatic nucleus with prominent nucleolus/ Tumor islands with dyskeratosis, increased abnormal mitotic figures and focal areas of necrosis. August 2004: second recurrence was treated surgically, but there wasn't histopathological description. January 2005: third local recurrence was treated only palliatively.	ki-67 (benign area- less than 1%/ malignant area- up to 42%), p53 protein +
Zhong et al. (2010)	CEOT with clear cells	Hybrid lesion: nests, cords and filaments of irregular polyhedral epithelial cells with clear cytoplasm and ample eosinophilic cytoplasm in a fibrous and eosinophilic stroma/ Hyaline material with frequent calcification, Congo red +/ Few mitotic figures (central portion of the tumor). Islands of polyhedral epithelial cells with nuclear pleomorphism and increased mitotic activity/ Microscopic foci of central central microcystic degeneration and necrosis in the squamous islands/ Periphery with discohesive areas of epithelial morphology alternating with areas of pseudoglandular appearance/ Bone and blood vessel invasion (peripheral portion of the tumor).	No recurrence	ki-67, 15% (malignant area) 3% (benign area), cytokeratin 19+, pancytokeratin+
Tabaksert et al. (2021)	CEOT with clear cells	Sheets of polyhedral squamous epithelial cells with abundant eosinophilic cytoplasm supported by fibrous stroma/ Eosinophilic matrix deposits with spherical calcifications in tumor islands/ Positive hyaline material for Congo red and apple-green birefringence/ Presence of clear cells/ Nuclear hyperchromatism and pleomorphism/ Scattered mitotic figures (benign CEOT).	After 4 years and 6 months: first recurrence exhibiting characteristics of malignity, with focal necrosis and perineural infiltration. Lung metastasis, with a central area of hyalinization and infarction, infiltrative sheets and islands of epithelial cells with similar characteristics to the primary lesion, pleomorphism and mitosis.	p40+, cytokeratin 5/6+, antigen CD56+, cytokeratin 7-, ki-67 (lung metastasis- over 20%)
Aoki et al. (2021)	--	Calcification in concentric rings of neoplastic epithelial cells with irregular deposition of scattered homogeneous eosinophilic masses, amyloid material (benign CEOT).	After 1 year: first recurrence with similar histological characteristics to the primary lesion, but with resorption of trabecular bone in some areas (benign CEOT). After 1 year and 3 months: second recurrence with slightly atypical tumor cells and scattered mitotic figures/ Tumor cells formed small nests within the stroma alongside extensive fibrogenesis, showing the invasive proliferation typical of squamous cell carcinoma/ Bone invasion and fracture (diagnosis of malignant transformation of CEOT to squamous cell carcinoma).	ki-67 (~40%), p53 protein +

Legend: CEOT (calcifying epithelial odontogenic tumor); IHC (immunohistochemistry). Source: Author.

**Table 3:** Therapeutic and prognostic aspects of CEOT cases with malignant transformation.

Authors (year of publication)	Treatment	Metastasis and location	Recurrence- Number	Follow-up period (months)	Outcome
Basu et al. (1984)	Surgery	Submandibular lymph node	Yes- 1	--	Remission
Veness et al. (2001)	Surgery and radiotherapy	Level III lymph node	Yes - 2	12	Remission
Cheng et al. (2002)	Surgery	--	No	10	Remission
Nagahama et al. (2002)	Surgery and chemotherapy	Lymph nodes and lung	Yes - 4	--	Remission
Kawano et al. (2007)	Surgery and chemotherapy	Lung	Yes - 3	--	Alive with disease
Demian et al. (2010)	Surgery, radiotherapy and chemotherapy	Roof and side wall of the orbit	Yes - 2	--	Death
Zhong et al. (2010)	Surgery	--	No	24	Remission
Tabaksert et al. (2021)	Surgery and radiotherapy	Lung	Yes - 1	60	Remission
Aoki et al. (2021)	Surgery	--	Yes - 2	27	Remission

Legend: CEOT (calcifying epithelial odontogenic tumor). Source: Author.

## Data Availability

The datasets used and/or analyzed during the current study are available from the corresponding author.
